# 

**Published:** 1802-12-01

**Authors:** 


### To CORRESPONDENTS.

*The Editors will be particularly obliged to their Correspondents reſiding in distant parts of the kingdom, if they will favour them with accounts of the prevailing Epidemics at this season of the year.*

END OF VOL. VIII.

---

[ W. Thorne, Printer, Red Lion Court, Fleet Street. ]

### INDEX.

ABERNETHY, Mr. on his operation for popliteal aneurifm 1
——————, his lectures announced 286
——————, on his mode of treating abceffes 206
Ablution, on the virtues of acetous 51
Abfcefs, on Mr. Abernethy's mode of treating 206
——————, a cafe of lumbar 44
Acharius, Dr. Eric, his cafe of pulmonary polypus 201
Acofta, anecdote of, 390
Adams's, Dr. defence of Mr. Hunter's phyfiological ideas and language 412
Affufion, obfervations on its ufe in typhus 158
——————, Mr. Pearfon's remarks on cold 357
Air, on purifying infected 184
——, on clearing apartments from noxious 575
Air-pump vapour bath, account of the 54
Alimentary canal, the ufe of alkalis in difeafes of the 293
Alkalis, their ufes in diforders of the ftomach and bowels 289
——————, their efficacy in difeafes of the alimentary canal 293
Allen's, Mr. lectures announced 286
Alpinus, his account of the effects of opium on the Egyptians 393
Anatomical plates of the bones and mufcles, reviewed 473
Anchylofis, obfervations on an univerfal, 284, 395
Aneurifm, cafes of (with a plate) 1
Angina Tracheali, a cafe of 203
Animal heat, obfervations on 500
—————— Oeconomy, on the effects of carbonous gas in 189
Animals, experiments and obfervations made on 522
Antiforic preparations, obfervations on 113
Ape, account of the inoculation of an 272
Apoplexia hydrocephalica, cafe of 62
Apoplexy, obfervations on 64, 77, 80
——————, Dr. Ryan's cafe of, with remarks 210
——————, Mr. Crowfoot in explanation on 116
——————, Dr. Langflow's Sketch of the controverfy on, reviewed 379
Argylefhire fencibles, account of the ophthalmia among the 185
Arm prefentation, Dr. Clark's letter on 394
Armiger, Mr. his anatomical demonftrations announced 383
Artificial minneral waters, obfervations on, 86, 488
Afphyxia, or apparent death, efficacy of Galvanifm in 256
Atkinfon, Mr. on the ufe of opium in fever 59
——————, on his cafe of apoplexy 65
Atmofphere, means of purifying the 529
Atmofpheric air, obfervations on the 573
Atracto-Ceros, account of a new genus of infects, fo named 282
Aveyron, account of the favage of 377
Babington's, Dr. lectures announced 286
Badger's, Mr. cafe of tinea capitis (with a plate 554
——————, Mr. on an eruptive difeafe 106
Bagdad, vaccine inoculation introduced at 195
Baillie's, Dr. feries of engravings on morbid anatomy reviewed 564
Barclay's, Dr. lectures announced 479
Barlow, Mr. on fhoulder prefentation 213
Barton, Dr. on the datura ftramonium 427
——————, on the fpigelia marilandica 428
——————, on vermifuge medicines 429
Bath, account of the difeafes at the Difpenfary of the city of 15, 207, 364
—— on the air pump vapour 54
Batty's, Dr. lectures announced 287
Beauvois, Cit. his account of a new genus of infects 282
Beddoes, Dr. his propofal of a fubfcription for Dr. Jenner 7
Bell's, Mr. anatomy of the brain, reviewed 473
——'s lectures announced 479
Bellamy, Dr. on the hooping cough 41
——————, on the zanthoxylon 453
Benevolent fociety of Herts and Effex, their refolution on the vaccine inoculation 192
Benzoic acid, experiments to difcover it in the urine of horfes 476
Berkeley, Earl of, his evidence on Dr. Jenner's petition 20
Bernard, Mr. on the ovarian dropfy (with a plate) 387
Berthollet, Cit. on the fulminating mercury 190
Beveridge, Mr. his cafe of uterine cancer 431
Bibb, Mr. on the modus operandi of medicines, reviewed 91
Bichat, profeffor, account of his works 382
Birch, Mr. his evidence on Dr. Jenner's petition 142
Black, Dr. his chemical lecture announced for publication 383
Blair, Mr. his fyftem of furgery announced 285
——————, his lectures announced 288
Blake, Mr. on the teeth in man and animals 558
Blegborough's, Mr. account of the air-pump vapour bath 54

Blegborough, Mr. on the ufe of cold water in typhus 158
———, on the gout 349
Blind, method of teaching them to write 572
Blizard, Meff. their lectures announced 383
Bones, on the difeafes of the 300
———, references to the, reviewed 279
Booker, Rev. Mr. his fermon on the cow-pox noticed 171
BOOKS, MEDICAL, CRITICAL ANALYSIS OF—Report to the National Inftitute of France refpecting the Artifical Mineral Writers, made at Paris, by N. Paul and Co. 86. Obfervations on the Arguments of Profeffor Rufh, in favour of the inflammatory Nature of the Difeafe produced by the Bite of a Mad Dog, by J. Meafe, M. D. 88. An Inquiry into the Modus Operandi of Medicines upon the Human Body, by W. W. Bibb, 90. Commentaries on the Hiftory and Cure of Difeafes, by William Heberden, M D. 179. Facts and Arguments on the Cow-pox, by Thomas Lee, 182. Account of an Ophthalmia which appeared in the fecond Regiment of Argylefhire Fencibles, with Obfervations on the Egyptian Ophthalmia, by A. Edmonftone, 185. Facts decifive in Favour of the Cow-pox, by R. J. Thornton, M. D. 185. A Treatife on the Means of purifying infected Air, of preventing Contagion, and arrefting it Progrefs, by L. B. Guyton Morveau, 185. A Fourth Differtation on Fever, by George Fordyce, M. D. 274. The Edinburgh School of Medicine, by W. Nifbet, M. D. 277. References to the Bones, by B. Simons, M. D. 279. Lectur s on Comparative Anatomy, tranflated from the French of G. Cuvier, by W. Rofs, 365, 466. Practical Obfervations on the Inoculation of Cow pox, by J. Bryce, 373 An Hiftorical Account of the Difcovery and Education of a Savage Man caught in the Woods near Aveyron, by E. M. Itard, 376. A Practical Synopfis of the Materia Medica, Vol. I. 379. An Hiftorical Sketch of the Controverfy on Apoplexy, &c. by R. Langflow, M. D. 379 A Synopfis and Explanation of the Chemical Nomenclature, by Samuel Mitchell, M. D. 380. The Medical Querift and Inveftigator, by W. P. Ruffel, 380. Obfervations on Pulmonary Confumption, by J. B. Regnault, 463. A Compendium of the Veterinary Art, by James White 466. A Treatife on the Morbid Affectionsof the Knee Joints, by James Ruffell, 470., Practical Information on the malignant Scarlet Fever and Sore Throat, by E. Peart, M D. 471. Practical Information on Inflammation of the Bowels, and ftrangulated Rupture, by the fame, 472. Practical Information on St. Anthony's Fire, or Eryfipelas, and on Erythematous Affections in general, by the fame, 473. Anatomical Plates of the Bones and Mufcles, by R. Hooper, M. D. 473. The Anatomy of the Brain, by Charles Bell, *ib.* On the Egyptian Ophthalmia, by Cit. Savarefi, 55[illegible] On the Teeth in Man and Animals, by Dr. Blake, 558. Collection of Papers to promote an Inftitution for the Preventiou of infectious Fevers, by Dr. Clark, 562 A Treatife on Brown's Syftem of Medicine, by H. Pfaff, M. D. 563. A Series of Engravings to illuftrate the Morbid Anatomy of fome of the moft important Parts of the Human Body, by M. Baillie, M. D. 564. Obfervations fur la Phthifie Pulmonaire, par J B. Regnault, M. D. *ib.* Practical Obfervations on Vaccination, by J. R. Coxe, M. D. 565. An Effay on the Structure and Formati n of the Teeth in Man and Animals, by R. Blake, M. D. 558
BOOKS, NEW, ANNOUNCED.—Dr. Stanger on the Neceffity and Means of fuppreffing Contagion in the Metropolis, 192. Mr Wilkinfon, of Sunderland, his Experiments and Obfervations on the Cortex Salicis Latifoliæ, *ib.* Mr. Blair's Syftem of Medical and Operative Surgery, 285. Dr. Wittman's Travels in Turkey, &c. *ib.* A Chemical Lecture of the late Dr. Black 383. A Courfe of Lectures on Zoonomia, by Dr. Garnett, 383. Dr. Walker's Obfervations on the Conftitution of Women, 480
Bofch's, Dr. defcription of a remarkable enlargement of the ovaria 444
Boudet's method of preparing phofphoric ether 569
Bourdeaux, prize queftion of the Medical Society at 280
Bowels, on the treatment of an inflammation of the 181
———, on the ufe of alkalis and abforbents in diforders of the 289
Bradley, Dr. a card to, from Dr. Pearfon, 156
———, his lectures announced, 287, 384
Bragge, Mr. his letter on the cow-pox 154
Brain, review of Bell's anatomy of the 473
Brandreth, Dr. his teftimonial in favour of vaccine inoculation 546
Breathing, cafe of a periodical difficulty of 401
Brenan, Mr. on pulmonary polypi 360

Brinkhurst, Mr. on the efficacy of alkalies in diforders of the ftomach and bowels 293
Brookes, Mr. his lectures announced 287
Brown, Dr. falls a victim to hypothefis 353
Bryce's, Mr. obfervations on the cow-pox reviewed 373
Buckholz's, Mr. method of preparing emetic tartar 92

Cadogan's, Dr. rules for gouty patients 355
Callifen, profeffor, on emetics in apoplexy 69
Cancer, cafes of uterine 431
Cancerous difeafes, obfervations on the treatment of 295
Canterbury, account of the hofpital at 123
Cantharides recommended in hydrophobia 89
Carbonous gas, on the efficacy of 189
Carpue, Mr. his lectures announced 287
Catalonia, fuccefs of vaccine inoculation in 573
Cataract, cafes of 115, 398
———, Mr. Leefon on 495
Chamberlaine, Mr. on the New Medicine Act 127
Chauffier, Cit. on the efficacy of the carbonous gas in animal œconomy 189
———, on preferving animal fubftances from putrefaction 479
———'s, Cit. experiments on animals with hydrogenous fulphurated gas 523
Chemical Nomenclature, on the new 550
Chemiftry, Dr. Prieftley on the new fyftem of 305
Chevalier Mr. his lectures announced 288
Chevalier's, Mr. cafe of puncturing the dura mater 505
Cholera, on the ufe of alkalis in 290
——— obfervations on 363
Church yards, on the inconveniencies of 224
Cicuta, its benefits in phthifis 85
Cinaber, obfervations on 573
Clarion, Cit. his defcription of a new fpecies of phaca 283
Clark's, Dr. Jofeph, letter on arm prefentation 394
Clarke's, Dr. lectures announced 287
Clarke, Dr. on preventing contagious Fevers, reviewed 562
Cline's, Mr. evidence on Dr. Jenner's petition 17
———, lectures announced 286
Cold water, obfervations on its ufe in typhus 158
———, Mr Pearfon on 356
———, Practicus on 442
———, on the advantages of in ulcers 206
Comparative Anatomy, Cuvier's lectures on, reviewed 365, 466
Conftantinople, progrefs of vaccination at 95
Confumptions, obfervations on 225
Contagion, on the means of preventing 186
Cooper's Mr. cafes of aneurifm (with a plate 1
Cooper's, Mr. lectures announced 286
Corvifart, Cit. on a fiftula in the ftomach 361
Cow-pox, Dr. Beddoes on 7
——— virus to be had gratuitoufly 568
——— nofological defcription of the *ib.*
———, Mr. Whalley on 26
———, Mr. Thomas on 165
———, on fuppofed unfuccefsful cafes of 169
———, extract from a fermon on 171
———, Lees facts and arguments on, reviewed 182
———, Facts decifive in favour of, reviewed 185
———, Mr. Jenkinfon on 197
———, Mr. Ring on cafes of 198
———, Bryce's obfervations on the inoculation of, reviewed 373
———, Steinbeck on that at Gottingen, in 1769 476
Coxe's, Dr. obfervations on vaccine inoculation, reviewed 565
Crichton's, Dr. lectures announced 287
Croft, Dr. his evidence on Dr. Jenner's petition 24
Crowfoot, Mr. on apoplexy in explanation 116
Cruickfhank's defence of the New Chemiftry, reply to 305
Cullen, Dr. on emetics in apoplexy 69
"Curiofities of common Water," extract from that Work 442
Curry, Dr. his lectures announced 286
———, elected phyfician to Guy's Hofpital 383
Cutaneous affections, fingular cafe of 508
Cuthbertfon, Mr. on diftinguifhing the two kinds of electricity 572
Cuvier's lectures on comparative anatomy, reviewed 364, 466
Cynanche tonfillaris, cafe of 15
Damart, M. his method of purifying vegetable oil 383
Dancer's, Mr. cafe of a negro turning white (with a plate) 97
Darracq, Cit. on the affinity of the earths with each other 474
Darwin, Dr. his advice to a gouty patient 355
Datura Stramonium, on the beneficial effects of the 426
Davies, Mr. obfervations on his cafes of apoplexy 65
Davis, Mr. J. B. his cafes and remarks on hydrocephalus internus 98
——— on difeafes of the liver 238

Deafnefs, effects of Galvanifm, on 324
Dean's, Mr. cafe of uterine cancer 432
De Carro, Dr his obfervations on the vaccine inoculation 193
Dennifon's, Dr. lectures announced, 287, 383
Diarrhœa, on the ufe of alkalis in 291
Diet, mode of, recommended to perfons weakened by mercury 11
Digitalis, its fuccefs in phthifis pulmonalis 82
Difeafes, on the endemic caufes of 221
———, on the effects of Galvanifm in 251
———, account of in the Bath Difpenfary 15, 207, 364
———, account of in an eaftern diftrict of London, from May 20, to June 20, 1802 14
———, from June 20, to July 20 112
———, from July 20, to Aug. 20 202
———, from Auguft 20, to September 20, 363
———, from September 20, to Oct. 20 396
———, from Oct. 20, to Nov. 20 496
———, Nifbet, Dr. on cancerous 294
Dogs, on the inoculation of 194
——, experiments on, by Dr. Viborg 271
Dolling's Mr. letter on the cow-pox 154
Drew's, Mr. letters on the cow-pox 153
Dropfy, cafe of an ovarian 387
Dupuyten, Mr. his account of a lufus naturæ 382
Dura mater, cafe of the fuccefsful puncture of the, 505
Dyfentery, cafe of 397
Dyfphagia, cafe of 35

Earth, on the nature of that which is eaten by the inhabitants of New Caledonia 191
Earths, on their combination with each other 474
Edinburgh, private Medical claffes at 479
————, School of Medicine, review of the 277
Edlin's, Mr. obfervations on meafles 28
Edmonftone's, Mr. account of an ophthalmia 185
Egypt, on the climate and difeafes of 449
Egyptian Ophthalmia, Cit. Savarefi on the 555
Ekeburg, Mr. his account of a new metallic fubftance 573
Electricity, difference between Galvanifm and 321
Electricity, on diftinguifhing the two kinds of 572
Emetics, on their effects in apoplexy 67, 79, 81
Emetic tartar, fhort and convenient method of preparing 92
Endemic difeafes, the caufes of 221
Eruptions, on puftulous 200
Eruptive difeafe, account of an 106
Eryfipelas, ufe of ammonia and aromatic confection in 473
Ether, new method of preparing phofphoric 569
Evidence on Dr. Jenner's petition 17, 141
———, obfervations on Dr. Pearfon's 265
Examen of electrical phænomena which do not appear to accord with the two fluids 477
Experiments on emetic tartar, by Mr. Buckholz 92
———— on carbonous gas, by Cit. Chauffier 189
————, on fulminating mercury 19
———— and obfervations on the cortex falicis latifoliæ announced 192
————, on Galvanifm in the cure of difeafes 250, 315
————, to prove that the fmall-pox is common to men and brutes 271
————, on frogs 338
————, on the affinity of the earths with each other, by Cit. Darracq 479
————, on the benzoic acid in the urine of horfes 476
————, electrical, by Cit Tremery 477
————, on the decolouration of vegetable liquors 478
———— Galvanic, applied to the ufe of Medicine 524
———— and obfervations on animals with the hydrogenous oxygenated gas 513
————, electrical 572, 573
———— on light and heat 572

Facts, and fome arguments refpecting inoculation of the cow-pox, by T. Lee, review of 182
—— decifive in favour of the cow-pox, by R J. Thornton, review of 185
—— concerning the efficacy of alkalis in difeafes of the alimentary canal 293
Farquhar, Sir Walter, letter to, on cancerous difeafes 295
Fens, ftate of thofe in Lincolnfhire 223
Fever, on the ufe of opium in 50
———, on the benefit of old Madeira in flow 89
———, on the ufe of cold water in 158
———, Dr. Fordyce's fourth differtation on, reviewed 274
———, on the affufion of cold water in 158, 357
———, on the mode of treating the puerperal 433
———, plan of an inftitution for preventing contagious 538
Fevers, account of peftilential and malignant 449

Finſbury Diſpenſary, report of ſurgical caſes in the 109, 203, 300, 398, 493

Fiſcher J. W. C. his method of preparing the red oxyd of mercury 573

Fiſtula, obſervations on one in the ſtomach 360

Fleming, Dr. on apoplexies, 69

Floyer, Sir John, a new edition of his Pſychrolufia, recommended 443

Fluids, objections to the theory of two electric 477

Forbes, Dr. of Edinburgh, teaches medical pupils the Latin language 576

Fordyce, Dr. review of his fourth diſſertation on fever 274

Fowler, Mr. on the extraction of teeth 119

Fox, Mr. his lectures announced 480

Frampton, Mr. his lectures announced 383

Francillon's, Mr. account of a new metal 573

Frogs, experiments on 338

Fulminating mercury, experiments on 190

Fumigation, account of the muriatic 187

Galen, on apoplexy 80

Galvanic experiments by Dr. Quenſel 524

Galvaniſm, application of, in a caſe of rheumatiſm 552

———, obſervations and experiments on (with plates) 250, 315

Gardner, Mr. his evidence on Dr. Jenner's petition 19

Garnett, Dr. death and character of 95

———, his lectures announced for publication 383

Gieſe, M. experiments to diſcover benzoic acid in the urine of horſes 476

Girdleſtone's, Dr. caſe of cutaneous affections 508

Glouceſter, account of the Infirmary at 124

———, jelly, account of the 83

Goe's, Mr. caſe of divided trachea 162

Gonorrhœa, form of an injection for 114

Gottingen, on the cow-pox at 476

Gout, beneficial effects of the vapour-bath in the 57

———, obſervations on the cauſe, nature, and cure of 349

———, relieved by cold bathing 400

Grapengieſſer, Dr. his obſervations and experiments on employing Galvaniſm in the cure of diſeaſes 250, 315

Griffiths, Mr. his evidence on Dr. Jenner's petition 24

———, Dr. mixture, account of an imitation of 48

Guerin, Cit. his deſcription of a new inſtrument for performing the operation of lithotomy (with a plate) 385

Guyton, Cit. confutation of his opinions on the affinity of earths 474

Guyton, his memoirs on the electric pi of Volta 479

Haighton's Dr. lectures announced 286

Hall's, Dr. communication on the fevers of the Levant 449

Harriſon, Dr. on the endemic cauſes of diſeaſes 221

Haſlewood, Mrs. obſervations on her caſe 228

Hauy, Cit. on teaching the blind to write 572

Headington, Mr. his lectures announced 383

Heart, caſe of an unuſual conformation of the 497

Heberden's, Dr. (ſen.) commentaries on the hiſtory of diſeaſes reviewed 179

———, Dr. (jun.) evidence on Dr. Jenner's petition 148

Her's, Dr. M. his oppoſition to vaccine inoculation 195

Heſſe, Dr. his zeal and ſucceſs in vaccine inoculation at Conſtantinople 95

Hippocrates, his practice of midwifry 214

———, character of 280

Hippocratic medicine, prize queſtion on 281

Hogben, Mr. on an early diſcharge of liquor amnii 260

Hooper's, Dr. lectures announced 287

———, anatomical plates of the bones and muſcles reviewed 473

Hooping cough, obſervations on 41

———, ſuperſeded by the ſmall-pox, caſe of 426

Horncaſtle, addreſs to the Medical Society at 221

Horſe, ſtones diſcovered in the inteſtinal canal of a 94

Horſes, on the greaſe in 193

———, experiments on the urine of 476

Hoſpitals, account of county 121

Howard, Mr. on his fulminating mercury 190

Human body, on the influence of the moon on the 401

Hunter, Mr. his phyſiological ideas and language defended 412

Hyacinthus non ſcriptus, analyſis of the bulbs of 475

Hydrocele, obſervations on 34

———, caſes of its cure by injections with a ſimple apparatus 175

Hydrocephalus internus, caſe and remarks on 98

Hydrogenous oxygenated gas, experiments with 513

Hydrophobia, obſervations on 88

Hypochondriacus affectus, caution relating to 180

Infanticide, obſervations on 447

Infection, on the means of preventing the progreſs of 529

Inflammation of the bowels, obfervations on 181
Injection, a form of one for gonorrhœa 114
———, by a fimple apparatus, fuccefsful in hydrocele 175
Inftruments, defcription of improved 482
Iron, on a blue oxydated 479
Irving, Mr. on vaccine inoculation 543
Itard, Dr. his account of the favage of Aveyron 376

Jamiefon, Dr. his lectures announced 384
Jardine, Mr. his defcription of new-invented furgical inftruments 482
Jaundice, cafes of puerperal 358
Jenkinfon, Mr. on the ufe of myrrh, fteel, &c. 48
———, his cafe of vaccine inoculation, 197

Jenner, Dr. propofal for rewarding 7, 164
———, evidence on his petition 17, 141
———, thanks voted to him by the Benevolent Medical Society 192
———, reflections on his remuneration, 196
———, vindication of, againft Dr. Pearfon 265
———, letter and prefent to, from the emprefs dowager of Ruffia 480
Jenner, Rev. Mr. his evidence on Dr. Jenner's petition 21
Jiggers, defcription of 455
Johnftone, Dr. firft employed muriatic acid in preventing contagion 186
Jordan, Mr. his evidence on Dr. Jenner's petition 18

Keate, Mr. (Surgeon General) his evidence on Dr. Jenner's petition 149
———, Mr. Robert, his evidence on the fame 150
Kent, account of the general hofpital for the county 123
Kidderminfter, on the malignant fever at 186
Kirby, Dr. J. his lectures announced 479
Knee Joint, treatife on the morbid affections of the, reviewed 470

Lacepede, Cit. on two fpecies of nondefcript oviparous quadrupeds 283

Langflow, Dr. on Dr. Jenner's remuneration 164

———, Mr. Crowfoot's reply to 116

———, his fketch of the controverfy upon apoplexy, reviewed 379

Lead, on the internal ufe of 209

Lectures, propofals for publifhing Dr. Garnett's 383

Lectures announced.—Mr. Cline and Mr. Cooper's on anatomy and furgery, 286. Drs. Babington and Curry on the practice of medicin , *ib.* Dr. Babington and Mr. Allen on chemiftry, *ib.* Dr. Curry on the theory of medicine and materia medica, *ib.* Dr. Haighton on midwifery and difeafes of women and children, *ib.* Ditto on Phyfiology, *ib.* Mr. Cooper on the principles and practice of furgery, *ib.* Drs. Roberts and Powel on the theory and practice of medicine, *ib.* Mr. Abernethy on anatomy, phyfiology, and furgery, *ib.* Mr. Macartney on comparative anatomy and phyfiology, *ib.* Dr. Powel on chemiftry and the materia medica, *ib.* Dr. Thynne on midwifry, and the difeafes of women and children, *ib.* Meffrs. Wilfon and Thomas on anatomy, phyfiology, pathology, and furgery, *ib.* Mr. Brookes on anatomy, phyfiology, and furgery, 287. Mr. Carpue on anatomy, *ib.* Dr. Hooper on the theory and practice of phyfic and morbid anatomy, *ib.* Dr. Crichton on the theory and practice of phyfic, chemiftry, and materia medica, *ib.* Dr. Bradley on the theory and practice of medicine, *ib.* Dr. Wells on the practice of phyfic, *ib.* Dr. Rowley on the ancient and modern medicine, *ib.* Drs. Ofborne and Clarke on midwifery, *ib.* Dr. Batty on the theory and practice of midwifery, *ib.* Dr. Dennifon and Squire on midwifery and the difeafes of women and children, *ib.* Mr. Blair on anatomy and animal oeconomy, 288. Mr. Pearfon on furgery, *ib.* Dr. Pole and Mr. Wagftaff on midwifery, and difeafes of women and children, *ib.* Mr. Taunton on anatomy, phyfiology, and furgery, *ib.* Mr. Chevalier on the principles and operations of furgery, *ib.* Meffrs. Headington and Frampton on anatomy, phyfiology, and furgery, 383. Meffrs. Blizard, &c. on furgery, *ib.* Dr. Dennifon on midwifery, *ib.* Dr. Jamefon's clinical, 384. Mr. Fox on the teeth, 480.

Lee, Thomas, facts and fome arguments refpecting inoculation of the cow-pox, reviewed 182

Leefon, Mr. on cataract 495

Lenticular, defcription of an improved 482

Le Roux, Cit. on a fiftula in the ftomach, 361

———, on the hyacinthus non fcriptus, 476

Leucoma, obfervations on 329

Levant, on the malignant and peftilential fevers of the 449

Lichen iflandicus, on the virtues of 568

Light and heat not the fame 573

———, cheap method of producing 572

Lincolnfhire, on the climate, foil, and difeafes of 221

Liquor amnii, cafe of the early difcharge of 29[illegible]

Lift of private medical claffes at Edinburgh 479
Lithotomy, defcription of a new inftrument for performing the operation of 385
Liver, on difeafes of the 238
London, account of difeafes in an eaftern diftrict of 14, 112, 202, 363, 396, 496
———, Inftitution for preventing contagious fever in 538
Longevity, queries concerning 381
Lowitz, M. on the decolouration of vegetables 471
Lumbar abfcefs, cafe of 44
Lufcombe, Mr. on ftyptic applications 197
Lufus naturæ, account of a fingular 382

Macartney, Mr. his lectures announced 286
Madeira, its ufe in typhus 89
Madnefs, remarkable cafes of 311
Magin's, Mr. cafe of cutaneous affection 508
Marcet, Dr. elected affiftant phyfician to Guy's hofpital 480
Marfhall, Dr. his evidence on Dr. Jenner's petition 22
Marfhes, prize queftion on deftroying the dangerous emanations from 478
Meafe's, Dr. obfervations on the arguments of profeffor Rufh concerning hydrophobia, reviewed 88
Meafles, obfervations on the 28
Medical Querift and Inveftigator reviewed 380
———, benevolent fociety, refolution of the 192
———, lectures.—See Lectures.
———, and phyfical intelligence 92, 189, 280, 381, 474, 595
———, and phyfical literature, critical retrofpect of 86, 179, 274, 365, 463, 555
Medicine, on the modus operandi of, reviewed 90
——— act, obfervations on the new 127
———, Galvanic experiments applied to the ufes of 524
Melia Azedarach, its ufe as a vermifuge 429
Mercury, on the infirmities arifing from the ufe of 9
———, on the ufes of borat of 11
———, analyfis of the fulminating 190
———, folution of oxygenated muriat of 479
Mercury, method of preparing the red oxyd of 573
Metal, account of a new 573
Metropolis, plan adopted for the prevention of contagious fever in the 538
———, Dr. Stanger's work on the means of fuppreffing contagion in the, announced 192
Michælis, Dr. on puerperal fever 433, 510
Mitchill, Dr. his fynopfis of chemical nomenclature, reviewed 380
Monro, Dr. opens a private diffecting room at Edinburgh 119
Moon, attempt to prove her influence on the human body 401
Morgan's, M. cafe of cataract 115
Morveau's, Cit. treatife on the means of purifying infected air, reviewed 185
———, on the means of purifying the atmofphere 529
Mofeley Dr. his evidence on Dr. Jenner's petition 141
Murray's, Mr. lectures announced 470
Myrrh, on the ufes of 48

Naples, vaccine inoculation fuccefsfully practifed in 23
Naphtha aceti martialis, preparation of 189
Nafh, Mr. T. his evidence on Dr. Jenner's petition 151
Negro, cafe of one turning white (with a plate) 97
New Caledonia, on the earth eaten by the inhabitants of 191
Nifbet, Dr. Edinburgh fchool of medicine reviewed 277
———, letter on cancerous difeafes 294
Nifmes, prize queftion by the inftitution of health at 477
Nomenclature, on the new chemical 550
Northampton, account of the infirmary at 122

Obfervations on the arguments of profeffor Rufh in favour of the inflammatory nature of the difeafe produced by the bite of a mad dog, reviewed 88
———, on Dr. Pearfon's evidence 265
———, on an univerfal anchylofis 395
———, on pulmonary confumption, reviewed 463
Œfophagus, defcription of an inftrument for 487
Okes, Mr. cafe of hooping cough fuperfeded by the fmall-pox 426
Oil, new method of purifying vegetable 383
Opiate frictions, cafe of its efficacy in fever 16
Ophthalmia, its prevalence in Egypt 450
Ophthalmia, Cit. Savarefi on the 555
Opium, on the ufe of in fever 50
———, on the modus operandi of 325, 388
———, on the fedative properties of 505
Ofborne's, Dr. lectures announced 287
Ofiander, profeffor, his cafe of delivery 95
Ovaria, on the enlargement of the 444
Ovarian dropfy, on the cure of (with a plate) 387

A

Oxford, account of the Radcliffe infirmary at 126

Palfy, on the ufe of the vapour-bath in 60
Paralyfis, beneficial effects of Galvanifm in 322
———, traumatica, cafe of 494
Parey, Ambrofe, account of 214
Paul, N. account of his artificial mineral waters 86
Pears, Mr. on fuppofed unfuccefsful cafes of vaccination 169, 547
Pearfon's, Dr. evidence on Dr. Jenner's petition 144
———, letter to Dr. Bradley 156
———, obfervations on the evidence of 265
———, Mr. on cold affufion in typhus 356
———, lectures announced 288
Peart, Dr. on the malignant fcarlet fever and fore throat reviewed 471
———, on inflammation of the bowels and ftrangulated rupture 472
———, on St. Anthony's fire, &c. 473
Percy, Cit. his obfervations on an univerfal anchylofis 284, 395
———, his cafe of extraordinary voracity 284
Pfaff, Dr. on Brown's Syftem of Medicine 563
Pfeiffer, Dr. on the cure of pulmonary confumption by falivation 308
Phaca, defcription of a new fpecies of 282
Phofphoric ether, new method of preparing 569
Phthifis pulmonalis, fuccefsfully treated with digitalis 82
Pitcairn, Dr. his advice to a hard drinker 553
Pictet, Cit. his experiments on light and heat 572
Plan for the prevention of contagious fever 538
Pneumatic apparatus, notice of a new 382
Pole, Dr. his lectures announced 288
Polyphagy, extraordinary inftance of 284
Poor, peculiarly liable to difeafes 110
Pope, Mr. his evidence on Dr. Jenner's petition 20
Portenfchlag, Dr. his zeal for vaccination 195
Powell's, Dr. lectures announced 286
Practical fynopfis of the materia medica reviewed 379
Prefentation, on fhoulder 213
———, Dr. Clarke on arm 394
Prieftley, Dr. his reply to Mr. Cruikfhank's defence of the new chemiftry 305
Prize queftion propofed by the Medical Society at Bourdeaux 380
Prize queftion propofed by the Inftitute of Health at Nifmes 479
Puerperal fever, Dr. Michaelis on 433, 510
Puerperal jaundice, cafes of 358
Pugnet, Dr. on the peftilential and malignant fevers of the Levant 449
Pulmonary confuinptions, obfervations on 463
Pulmonary confumption cured by falivation 308
Pulmonary polypi, cafe of 360
——— polypus, cafe of a 201
Pulteney's, Dr. letter to Dr. Pearfon 153
Puftulous eruptions, obfervations on 200

Quadrupeds, on two fpecies of non defcript oviparous 283
Quackery, on the evils of 246
———, on the means of fuppreffing 461
Quenfel's, Dr. Galvanic experiments applied to the ufe of medicine 524
Queftions, by Sir John Sinclair, on longevity 381

Rafpatory, defcription of an improved 483
Read, Mr. his pneumatic apparatus noticed 382
Reece, Mr. on the lichen Iflandicus 564
References to the bones for the ufe of anatomical fchools reviewed 279
Regnault's, Dr. obfervations on pulmonary confumption reviewed 463
———, Dr. obfervations fur la Phthifie Pulmonaire, reviewed 564
Reid, Dr. his tranflation of Dr. Itard's account of a favage man 376
Rheumatifm, cafe of acute 59
———, on the fuccefs of Galvanifm in 552
Ricards, Mr. his report of furgical cafes at the Finsbury Difpenfary 108, 203, 300, 398, 493
Ring, Mr. his obfervations on antiforic preparations 113
———, on Mr. Pears's cafes 198
———, on puftulous eruptions 200
Roberts, Dr. his lectures announced 286
Robertfon, Mr. on hydrocele 175
Robifon, Prof. announces a chemical lecture of Dr. Black's for publication 383
Rogers, Mr. on digitalis in phthifis pulmonalis 82
Rofs, Mr. his tranflation of Cuvier's lectures on comparative anatomy 365, 463
Rowlands, Mr. his obfervations on the hydrocele 34
Rowley's, Dr. evidence on Dr. Jenner's petition 142
———, lectures announced 187
Ruffel, Dr. on the ufe of opium among the Turks 326
———, W. P. his Medical Querift rereviewed 389

Ruſſel, Mr. his morbid affections of the knee joint reviewed 470

———, his lectures announced 479

Ruſſia, letter to Dr. Jenner from the empreſs dowager of 480

Ryan, Dr. obſervations on the internal uſe of lead 209

Salivation, caſe of pulmonary conſumption cured by 308

Salmon, Mr. on clearing apartments from noxious air 575

Saunders, Dr. on artificial mineral waters 488

Savareſi, Cit. on the Egyptian Ophthalmia 555

Savigny's, Mr. new invented ſurgical inſtruments deſcription of (with engravings) 481

Scarlatina anginoſa, prevalence and deſcription of 496

Sebald, Mr. diſcovers a quantity of ſtones in the inteſtinal canal of a horſe 94

Sequin's, Cit. obſervations on cinnaber 573

Seſamum, Dr. Spence on 553

Sheep, on the tubercles in their lungs 226

Sheep-pox, obſervations on 272

Shoulder preſentation, obſervations on 213

Simons's, Dr. references to the bones reviewed 279

Simpſon, Mr. his evidence on Dr. Jenner's petition 25

Sims, Dr. letter from Dr. Clark of Dublin to 394

Sinclair Sir John, his queſtions on longevity 381

Skey, Dr. his evidence on Dr. Jenner's petition 24

Small-pox, experiments to prove it common to men and brutes 271

———, caſe of hooping cough ſuperſeded by the 426

Spence, Dr. on the ſeſamum 553

Spigelia Marilandica, its beneficial effects in caſes of worms 428

Squires, Dr. his lectures announced 287

Stanger, Dr. announces a work on the means of ſuppreſſing contagion 192

Steel, on the uſes of 48

Steinbeck, Mr. on the cow-pox, at Gottingen, in 1769 476

Stevenſon's Dr. caſe of Dyſphagia 35

Stomach, on the uſe of alkalis and abſorbent in diſorders of the 290

———, on a fiſtula in the 361

Stumps, deſcription of an inſtrument for extracting 484

Styptic applications, remarks on 197

Surgical caſes in Finſbury Diſpenſary, report of 108, 203, 300, 493

Surgical inſtruments, deſcription of new invented 482

Tartar, new method of preparing emetic 92

Taunton's Mr. lectures announced 288

Taylor, Mr. his evidence on Dr. Jenner's petition 18

Teed, Mr. on the application of Galvaniſm in rheumatiſm 552

Teeth, on the extraction of the 110

———, on the ſtructure of the 558

Thomas, Mr. on vaccine inoculation 105

———, Mr. W. his lectures announced 268, 384

Thomſons, Dr. lectures announced 479

Thornton's, Dr. evidence on Dr. Jenner's petition 19

——— Facts deciſive, in favour of the Cow-pox, reviewed 185

Throat, caſe of a tumour of the 495

Tinea capitis, Mr. Badger's caſe of 554

Tobacco, its uſe as a vermifuge 428

Trachea, caſe of divided 162

Tranzieri's, Dr. caſe of a periodical difficulty of breathing, and on the influence of the Moon 401

Tremery's, Cit. examen of electrical phenomena 477

——— his electrical experiments 573

Trephine and Trevet, deſcription of the 484

Tupper's, Mr. hiſtory of a girl with an extraordinary conformation of the heart 497

——— obſervations on animal heat 500

Turks, ſucceſs of vaccine inoculation among the 96

Tynicola on apoplexy 64

Typhus, on the uſe of opium in 51

——— on the uſe of cold water in 158

——— Mr. Pearſon on cold affuſion in 356

Tyro on apoplexy 77

Ulcers, obſervations on 205

Urine, experiments on that of horſes 476

Uterine cancer, two caſes of 431

Vaccination, on ſuppoſed unſucceſsful caſes of 169

Vaccination, obſervations on 565

Vaccine inoculation, Mr. Whalley on 26

———, its ſucceſs in Turkey 95

———, Mr. Thomas on 165

———, Reſolution of the Benevolent Medical Society, concerning 192

———, Dr. De Carro on 194

———, Mr. Jenkinſon on 197

———, Mr. Ring on 198

———, letter from the empreſs dowager of Ruſſia on 480

———, Dr. Brandreth's teſtimonial in favour of 546

———, Dr. Waterhouſe on 543

Vaccine inoculation, Mr. Irving on 543
——, its success in Catalonia 573
Vaccine Pock Inftitution, account of the 565
Vage, Dr. on the treatment of the venereal difeafe 7, 172
Vapour bath, on the air pump 54
Vaughan, Dr. W. on Mrs. Haflewood's cafe 230
——, Dr. J. his two remarkable cafes of madnefs 311
Vauquelin, Cit. on the nature of the earth which is eaten by the inhabitants of New Caledonia 191
——, his account of a blue oxydated iron 479
Vegetable oil, new method of purifying 383
Vegetables, on the decolouration of 477
Venereal difeafe, criticifms on the treatment of 7, 172
Vermifuge medicines, obfervations on 428
Veterinary art, review of, a compendium of the 466
Viborg, Prof. his experiments relative to the fmall-pox 271
Volta, Refearches on the electric pile of 478
Voracity, cafe of extraordinary 284

Walker's, Dr. obfervations on the conftitution of women announced 480
Walker, Mr. his cheap method of producing light 573
Ward, Mr. on the modus operandi of opium 325, 388
Water, cold, on its ufe in typhus 158
—— in ulcers 206
—— in burns and fcalds 443
——, on the affufion of 356
Waterhoufe, Dr. on the cow-pox 543
Waters, on artificial mineral 488
Wells, Dr. his lectures announced 287
Whalley, Mr. on vaccine inoculation 26
White, Mr. W. on the efficacy of opiate friction in fever 16
——, James, compendium of the veterinary art reviewed 466
Wilkinfon, Mr. his work on the cortex falicis latifoliæ announced 192
Wilfon's Mr. cafe of lumbar abfcefs 44
——, lectures announced 286
Wittman's, Dr. travels in Turkey announced 285
Wolf, Dr. his abominable conduct to difcredit vaccination 195
Woodforde, Dr. his cafe of apoplexia hydrocephalica 62
Woodhoufe, Dr. letters to him from Dr. Prieftley 305

York, account of the hofpital at 122
——, duke of, withdraws his name from the vaccine Inftitution, and why 267

Zanthoxylon, Dr. Bellamy on the virtues of the 453
Zezegery, account of the 553
Zoonomia, Dr. Garnett's lectures on announced 383

### REFERENCES TO THE PLATES.

Mr. Cooper's mode of tying an Artery — — — — 1
Mr. Dancer's cafe of a Negro turning white — — — 97
Medical applications of Galvanifm, plate 1 and 2 250, 315
Guerin's porte conducteur, and Mr. Bernard's inftrument 385
Mr. Savigny's new Surgical inftruments, plate 1 and 2 — 482
Mr. Magin's cafe of Cutaneous Affection — — — 509
Mr. Badger's cafe of Tinea Capitis — — — — — 554

